# From Compassion to Satisfaction: The Role of Body Appreciation in the Relation Between Flows of Compassion and Couple Satisfaction in Postpartum Women

**DOI:** 10.3390/healthcare14142236

**Published:** 2026-07-22

**Authors:** Cristian Di Gesto, Camilla Matera, Amanda Nerini, Chiara Rollero, Daniela Caso, Anna Rosa Donizzetti, Caterina Grano

**Affiliations:** 1 Department of Psychology, Sapienza University, 00185 Rome, Italy; caterina.grano@uniroma1.it; 2 Department of Education, Languages, Intercultures, Literatures and Psychology, University of Florence, 50135 Florence, Italy; camilla.matera@unifi.it (C.M.); amanda.nerini@unifi.it (A.N.); 3 Department of Psychology, University of Turin, 10124 Turin, Italy; chiara.rollero@unito.it; 4 Department of Humanities, University of Naples “Federico II”, 80138 Naples, Italy; daniela.caso@unina.it (D.C.); donizzet@unina.it (A.R.D.)

**Keywords:** postpartum women, compassion flows, body image, body appreciation, couple satisfaction

## Abstract

**Background/Objectives:** The postpartum period involves profound bodily, psychological, and relational changes that may challenge women’s well-being and couple functioning, yet the role of flows of compassion and positive body image in postpartum couple satisfaction remains largely unexplored. The present study examined whether body appreciation mediated the associations between different flows of compassion (self-compassion, compassion toward others, and compassion from others) and couple satisfaction in postpartum women. **Methods:** A total of 230 Italian postpartum women who had given birth in the previous six months (Mage = 33.65, SD = 4.43) completed online self-report measures assessing self-compassion, compassion toward others, compassion from others, body appreciation, and couple satisfaction. Direct and indirect associations among compassion flows, body appreciation, and couple satisfaction were examined using path analysis, controlling for breastfeeding status. **Results:** Self-compassion and compassion from others were positively associated with body appreciation, which in turn was positively associated with couple satisfaction; compassion from others also showed a direct positive association with couple satisfaction, and significant indirect effects of self-compassion and compassion from others on couple satisfaction through body appreciation were observed. **Conclusions:** Body appreciation may represent a key psychological mechanism through which compassion-based processes support relational well-being in the postpartum period. This study is the first to examine the mediating role of body appreciation in the relationship between compassion flows and couple satisfaction in postpartum women. Integrating compassion flows with positive body image advances understanding of how intrapersonal and interpersonal emotional resources contribute to couple satisfaction following childbirth and informs interventions aimed at supporting postpartum adjustment.

## 1. Introduction

The postpartum period represents a profound transition in women’s lives, characterized by marked physical, psychological, and relational changes. Following childbirth, women experience enduring bodily modifications, shifts in identity, and the reorganization of intimate relationships, often accompanied by heightened vulnerability to psychological distress and relational strain [1]. Although this period is frequently portrayed as a time of fulfilment and bonding, empirical evidence consistently indicates that the first months after birth are associated with declines in couple satisfaction, disruptions in romantic relationships, and increased body-related concerns [2,3].

Couple satisfaction refers to the subjective evaluation of the extent to which one’s romantic relationship meets personal needs, expectations, and desires, reflecting the individual’s overall sense of fulfilment within the relationship. Although closely related to broader constructs such as relationship quality and relationship adjustment, couple satisfaction specifically captures individuals’ affective appraisal of their relationship, whereas relationship quality encompasses multiple relational dimensions (e.g., communication, intimacy, conflict, and support), and relationship adjustment refers to the couple’s ability to adapt to relational challenges and developmental transitions [2].

Pregnancy and childbirth often result in women’s bodies diverging from prevailing sociocultural appearance ideals, exposing new mothers to heightened self-consciousness and external pressures to rapidly “return” to a pre-pregnancy body [4,5,6]. Qualitative and quantitative studies consistently show that postpartum women frequently report body dissatisfaction, ambivalence toward their bodies, and tension between appreciation for bodily functionality and dissatisfaction with appearance-related changes [3,5,7].

Body image encompasses individuals’ perceptions, thoughts, and feelings about their physical appearance and bodily functioning [8]. During the postpartum period, body image becomes not only an individual but also a relational experience. Research has shown that women’s body-related concerns are associated with reduced comfort with intimacy, lower sexual satisfaction, and poorer relationship quality after childbirth [9,10]. Conversely, more positive and accepting experiences of one’s postpartum body may foster emotional and physical closeness within the couple [11].

Recent advances in body image research emphasize positive body image as a multidimensional construct that extends beyond the mere absence of body dissatisfaction [12,13]. One of its core dimensions is body appreciation, defined as accepting, respecting, and valuing one’s body for its functionality and uniqueness while resisting sociocultural appearance pressures [14]. Body appreciation has demonstrated strong measurement invariance across genders, age groups, and sociocultural contexts, supporting its role as a robust indicator of positive body image [15]. Emerging evidence further suggests that greater body appreciation is associated with reduced body-related self-consciousness, greater comfort with intimacy [16], more positive sexual experiences with one’s partner [17], and greater romantic relationship satisfaction through sexual esteem and sexual assertiveness [18]. Qualitative findings also indicate that women may simultaneously experience appearance-related dissatisfaction while developing greater appreciation for their bodies’ functionality and resilience [5]. Collectively, these findings suggest that body appreciation is a potential protective factor for relational functioning during the postpartum transition. Among the various dimensions of body image, body appreciation emerges as a particularly relevant construct because it represents a core component of positive body image and is conceptually aligned with compassion-based models, both of which emphasize acceptance, kindness, and respect toward oneself rather than merely reducing body-related distress. Furthermore, although the postpartum period is frequently associated with body dissatisfaction, qualitative research suggests that it may also foster a growing appreciation of the body’s functionality, resilience, and capacity following pregnancy and childbirth [5]. As such, body appreciation represents a particularly relevant psychological resource through which compassion may promote couple satisfaction during this important developmental transition.

### 1.1. Compassion as a Psychosocial Resource

Compassion has been conceptualized as sensitivity to suffering, accompanied by a motivation to alleviate and prevent it [19]. Contemporary models distinguish between different flows of compassion, including compassion directed toward the self (self-compassion), compassion expressed toward others, and compassion received from others [20]. These flows are theoretically distinct and may differentially influence self-regulation, emotional well-being, and interpersonal functioning. Compassion from others refers to the capacity to receive and feel supported by caring responses from significant people in one’s social environment, including but not limited to intimate partners, family members, friends, and other relevant figures [20].

The empirical literature on the relationship between compassion and couple satisfaction has predominantly focused on self-compassion. Self-compassion has been associated with greater emotional regulation, lower distress, and more adaptive coping across a range of populations [21,22,23]. Higher self-compassion has been linked to greater sexual satisfaction and lower sexual dissatisfaction in community samples of long-term couples [24], as well as to lower sexual distress in clinical populations [25].

Despite these contributions, important gaps remain in the literature.

First, existing studies have paid little attention to compassion flows other than self-compassion, such as compassion from others and compassion toward others. Although constructs related to partner-directed compassion (e.g., compassionate love) have been examined in specific contexts, such as reproductive loss, where they appear to be more strongly associated with relationship and sexual satisfaction than self-compassion [26], these constructs are conceptually distinct from compassion flows as defined in integrative compassion models and have not been examined alongside self-compassion within a unified framework.

Second, much of the available evidence derives from non-postpartum samples, including middle-aged married partners [27], or clinical populations [28]. When perinatal contexts are considered, findings are mixed, particularly with respect to the stability of associations between compassion-related processes and sexual or couple satisfaction across the transition from pregnancy to the postpartum period. For example, preliminary dyadic research suggests that associations between self-compassion and sexual satisfaction observed during pregnancy may not extend into the postpartum period [24,26], indicating that compassion-related processes may operate differently during this unique developmental transition.

Finally, studies examining compassion and couple or sexual satisfaction have rarely integrated body image, and virtually none have considered positive body image as a relevant psychological construct in the association between compassion and couple satisfaction.

Theoretical perspectives grounded in affect regulation propose that compassion activates affiliative and soothing systems that counteract threat-based self-criticism and shame [19]. From this perspective, self-compassion and receiving compassion from others may foster a kinder, more accepting relationship with the body, supporting body appreciation in the face of postpartum changes. Experimental and correlational findings support this view, showing that compassion-based processes are closely linked to positive body image [12,29]. Specifically, in the general population, Matera et al. (2025) [29] showed that brief compassion-based writing tasks targeting self-compassion, compassion from others, and compassion toward others produced significant increases in body appreciation compared with a control condition, highlighting the relevance of all three compassion flows for fostering a respectful and accepting relationship with the body.

Despite the theoretical plausibility of this pathway, no empirical studies to date have examined whether body appreciation mediates the relationship between compassion and couple satisfaction among postpartum women, nor whether such processes operate across different compassion flows.

### 1.2. The Current Study

The present study aimed to address these gaps by examining the relationships between different flows of compassion (i.e., self-compassion, compassion from others, and compassion toward others), body appreciation, and couple satisfaction in a sample of postpartum women. Specifically, the study seeks to explore both the direct associations between compassion flows and couple satisfaction and the indirect associations operating through body appreciation.

By integrating compassion-based processes, positive body image, and couple satisfaction, this study seeks to advance understanding of the psychosocial mechanisms that support couple well-being during a critical, understudied life transition. The findings may inform both theory and intervention by identifying compassion-related pathways through which body appreciation contributes to relational satisfaction in the postpartum period.

Accordingly, we hypothesized that higher levels of self-compassion, compassion toward others, and compassion from others would be positively associated with body appreciation and with couple satisfaction (Hypothesis 1).

It was hypothesized that body appreciation would be positively associated with couple satisfaction (Hypothesis 2) and that it would mediate the associations between the three flows of compassion and couple satisfaction (Hypothesis 3), thereby identifying body appreciation as a key psychological mechanism linking compassion processes to relational well-being.

Finally, breastfeeding status was included in the model as an additional postpartum experience potentially associated with couple satisfaction. Breastfeeding involves substantial changes in daily routines, partner involvement, and emotional support, which may influence couple interactions and perceived relationship quality. Therefore, breastfeeding represents a relevant contextual variable when examining individual differences in couple satisfaction among postpartum women [30,31].

**Figure 1:**
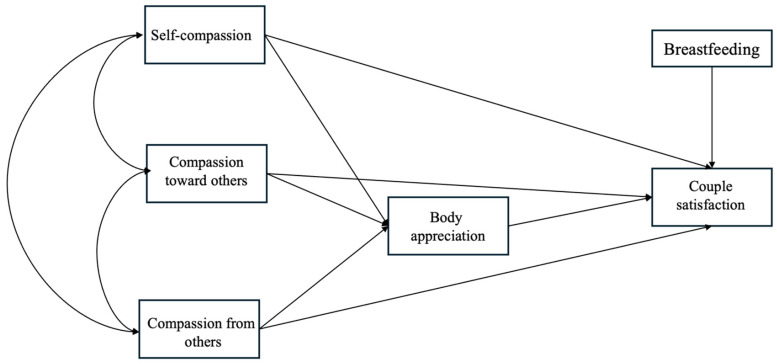
Hypothesized model.

The hypothesized model is displayed in Figure 1.

## 2. Methods

### 2.1. Participants

The sample consisted of 230 postpartum women. Participants ranged in age from 20 to 44 years (*M* = 33.65, *SD* = 4.43). All participants were within six months postpartum at the time of data collection.

No cases were excluded due to missing data, and all 230 participants were included in the analyses. Sociodemographic details and perinatal characteristics are provided in Table 1.

### 2.2. Procedure

Participants were recruited through collaborations with maternity and birth centres across Italy, private maternity services, community-based perinatal networks, and social media. Recruitment followed an opportunistic sampling strategy. Eligibility criteria required participants to (a) be at least 18 years old, (b) identify as women, (c) be in the postpartum period within six months after childbirth, and (d) be able to speak and understand Italian fluently.

All participants were informed that their participation was voluntary, anonymous, and confidential, and that they could withdraw from the study at any time without providing justification. Prior to accessing the questionnaire, participants were required to provide digital informed consent. This was facilitated through a web link that directed eligible participants to a secure online survey hosted on a platform specifically developed for the present research project.

The introductory page of the survey outlined the study’s objectives, inclusion criteria, confidentiality safeguards, and contact details of the research team. Only participants who confirmed that they met the inclusion criteria and provided informed consent were allowed to proceed. The questionnaire first included items assessing sociodemographic characteristics and postpartum-related information, followed by a battery of standardized self-report measures assessing compassion flows, body appreciation, and couple satisfaction.

No financial or material compensation was offered for participation. Completion of the questionnaire required approximately 15 min.

The study was approved by the Ethics Committee of the university with which some of the authors are affiliated (prot. n. 0639477, 7 December 2023). All procedures were conducted in accordance with the ethical standards of the institutional and/or national research committee and with the 1964 Helsinki Declaration and its subsequent amendments or comparable ethical guidelines. This research was funded by the Italian Ministry of University and Research (MUR), PRIN 2022 NextGenerationEU (Grant No. B53D23019650006).

### 2.3. Measures

Sociodemographic and Perinatal Characteristics. Sociodemographic information including age, education level, marital status, nationality, place of residence, and childbirth-related information including parity and breastfeeding were assessed.

Flows of Compassion. The Italian version [32] of the Compassionate Engagement and Action Scales (CEAS) [20] was used to assess different flows of compassion. The CEAS measures compassion across three distinct dimensions—self-compassion, compassion toward others, and compassion from others—rated on a Likert scale ranging from 1 (never) to 10 (always), with higher scores indicating greater levels of compassion in each respective flow. Self-compassion was assessed through 10 items reflecting individuals’ ability to engage with and respond constructively to their own suffering (e.g., “I think about and find helpful ways to deal with my suffering”). Compassion toward others was measured with 10 items assessing sensitivity to and meaning-making of others’ suffering (e.g., “I reflect on and try to make sense of other people’s suffering”). Compassion from others was evaluated using 10 items capturing the perception of receiving compassion and emotional responsiveness from others (e.g., “Others are emotionally moved by my suffering”). Each flow was assessed using two complementary components: compassionate engagement, which refers to sensitivity and openness to suffering (e.g., noticing distress, being emotionally moved by it, and tolerating related discomfort), and compassionate action, which refers to the motivation and ability to take helpful steps to alleviate or prevent suffering (e.g., thinking about what may be helpful and acting to reduce suffering).

In the present sample, internal consistency was good for all three subscales, with Cronbach’s *α* coefficients of 0.86 for self-compassion, 0.87 for compassion toward others, and 0.88 for compassion from others.

Body Appreciation. The Italian version [33] of the Body Appreciation Scale–2 (BAS–2) [12] was used to assess participants’ body appreciation. The scale consists of 10 items (e.g., “I feel good about my body”) rated on a 5-point Likert scale ranging from 1 (never) to 5 (always). Higher scores indicate greater levels of body appreciation. In the present study, the scale’s internal consistency was excellent (Cronbach’s *α* = 0.96).

Couple Satisfaction. Couple satisfaction was assessed using the Dyadic–Familial Relationship Satisfaction Scale (DFRS) [34]. The scale consists of 9 items evaluating satisfaction with different aspects of the couple relationship. Items are rated on a 5-point Likert scale ranging from 0 (not at all satisfied) to 4 (completely satisfied), with higher scores indicating greater couple satisfaction. An example item is “How satisfied are you with the way your needs and desires are met in your current relationship?” In the present study, the internal consistency of the scale was good (Cronbach’s *α* = 0.96).

### 2.4. Data Analyses

First, descriptive statistics and bivariate correlations among all study variables were computed. Subsequently, a path analysis model was tested in which the three flows of compassion (self-compassion, compassion toward others, and compassion from others) were specified as antecedents of body appreciation, which in turn was specified as a predictor of couple satisfaction. Breastfeeding status was included in the model as a control variable.

The dataset contained no missing values; therefore, no data imputation procedures were required.

The three compassion variables were allowed to covary, consistent with the theoretical framework proposing that compassion flows are distinguishable yet interrelated dimensions of compassion [20]. Modeling these covariances allowed the estimation of the unique associations of each compassion flow with body appreciation while accounting for their shared variance. Prior to model estimation, assumptions for path analysis were evaluated. Specifically, the distributions of the study variables were examined through skewness and kurtosis values, which indicated acceptable univariate normality [35]. Bivariate correlations and covariance estimates supported the specification of the proposed model, and no evidence of problematic multicollinearity was observed [36]. The hypotheses were tested using AMOS [37] (version 29). Indirect effects were estimated using a bootstrapping procedure to assess the presence and magnitude of mediation effects [38].

The sample size exceeded the recommended minimum of 200 participants for path analysis [39]. Parameter estimates were obtained using the maximum likelihood method. Model fit was evaluated using multiple goodness-of-fit indices: the χ^2^/df ratio, with values of 2 or lower indicating good fit; the Comparative Fit Index (CFI); the Tucker–Lewis Index (TLI); the Incremental Fit Index (IFI), with values above 0.95 indicating good fit; the Normed Fit Index (NFI), with values above 0.90 considered acceptable; the Root Mean Square Error of Approximation (RMSEA), including its 95% confidence interval (RMSEA 95% CI); and the Standardized Root Mean Square Residual (SRMR). Consistent with commonly adopted recommendations, RMSEA and SRMR values ≤ 0.08 were interpreted as indicating acceptable model fit, whereas lower values reflect a closer approximation between the hypothesized model and the observed data [40]. Statistical significance was evaluated using a two-tailed alpha level of 0.05.

## 3. Results

### 3.1. Descriptive Statistics and Bivariate Correlation

Table 2 presents descriptive statistics (means and standard deviations) and intercorrelations among flows of compassion (i.e., self-compassion, compassion toward others, compassion from others), body appreciation, and couple satisfaction. The data were normally distributed (skewness < 1.60; kurtosis < 4.19), as the skewness for all variables was lower than 2 and the kurtosis was lower than 7 [35].

As shown in the correlation matrix, self-compassion was strongly correlated with compassion toward others (*r* = 0.71) and moderately correlated with compassion from others (*r* = 0.48). Compassion toward others and compassion from others were also positively correlated (*r* = 0.35). Body appreciation was positively associated with self-compassion (*r* = 0.46), compassion toward others (*r* = 0.33), and compassion from others (*r* = 0.39). Couple satisfaction showed significant positive associations with self-compassion (*r* = 0.41), compassion toward others (*r* = 0.36), compassion from others (*r* = 0.49), and body appreciation (*r* = 0.40). All correlations were statistically significant (*ps* < 0.001).

### 3.2. Path Model

**Figure 2:**
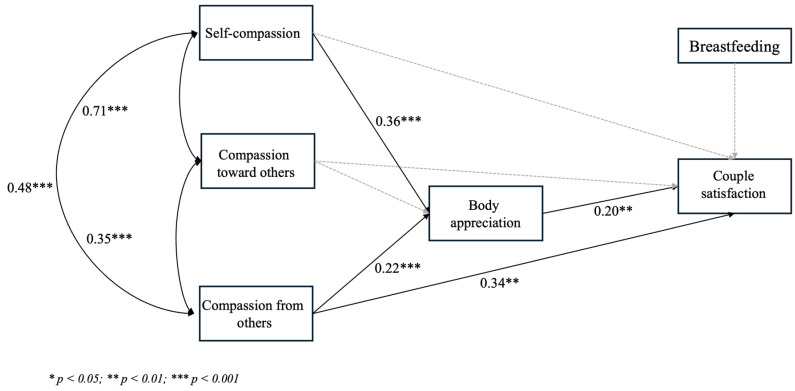
Observed model.

The hypothesized model was tested using path analysis to examine the direct and indirect associations among the three flows of compassion (self-compassion, compassion toward others, and compassion from others), body appreciation, and couple satisfaction. The model (Figure 2) showed a good fit to the data, *χ^2^*_(4)_ = 7.96, *p* = 0.09, NFI = 0.98, IFI = 0.99, TLI = 0.96, CFI = 0.99, RMSEA = 0.06 (95% CI [0.000, 0.133]), *p* = 0.28, SRMR = 0.03. Covariances among the three compassion dimensions ranged from 0.35 (*p* < 0.001) to 0.71 (*p* < 0.001).

Hypothesis 1 was partially supported. Self-compassion (*β* = 0.36, *p* < 0.001) and compassion from others (*β* = 0.22, *p* < 0.001) were positively and significantly associated with body appreciation, whereas compassion toward others was not directly related to this outcome variable.

In line with Hypothesis 2, body appreciation was positively and significantly associated with couple satisfaction (*β* = 0.20, *p* < 0.01). In addition, compassion from others showed a significant direct association with couple satisfaction (*β* = 0.34, *p* < 0.01), whereas self-compassion and compassion toward others were not directly related to couple satisfaction. Breastfeeding status was retained as a control variable.

Regarding indirect effects (Hypothesis 3), bootstrapping analyses indicated that self-compassion was indirectly associated with couple satisfaction through body appreciation (standardized indirect effect = 0.071, 95% CI [0.027, 0.159], *p* = 0.002). A significant indirect effect was also found for compassion from others on couple satisfaction via body appreciation (standardized indirect effect = 0.043, 95% CI [0.015, 0.100], *p* = 0.003). By contrast, the indirect effect of compassion toward others on couple satisfaction through body appreciation was not significant (standardized indirect effect = 0.000, 95% CI [−0.039, 0.046], *p* = 0.924).

Table 3 presents the indirect effect values from the path analysis.

The model explained 32% of the variance in couple satisfaction and 25% of the variance in body appreciation [41].

## 4. Discussion

The postpartum period is a major life transition marked by substantial physical, psychological, and relational changes [1]. In this phase, women often face enduring bodily modifications and increased exposure to body-related concerns, while couple dynamics may concurrently undergo adjustment and potential strain [2,3]. Building on these premises, the present study examined how the three flows of compassion—self-compassion, compassion toward others, and compassion from others [20]—relate to body appreciation and couple satisfaction among postpartum women, and whether body appreciation was associated with the observed relationships between compassion flows and couple satisfaction among postpartum women.

Overall, the findings support the relevance of compassion flows and positive body image for understanding relational adjustment in the postpartum period. The observed model showed that self-compassion and compassion from others were positively associated with body appreciation, whereas compassion toward others was not directly related to this outcome. In turn, body appreciation was positively associated with couple satisfaction. Compassion from others also showed a direct association with couple satisfaction. Finally, statistically significant indirect associations through body appreciation were observed for self-compassion and compassion from others in relation to couple satisfaction, whereas no significant indirect association emerged for compassion toward others.

The positive association between self-compassion and body appreciation is consistent with theoretical models suggesting that compassion toward the self activates affiliative and soothing regulatory systems that counteract threat-based self-criticism and shame [19].

The findings are also consistent with previous research showing that compassion-based processes are associated with higher body appreciation and lower appearance-related self-criticism [12,21] and extend previous experimental findings from the general population indicating that compassion-focused interventions can enhance body appreciation among women [29]. In the postpartum period, women experience visible bodily changes and heightened self-consciousness, often within a sociocultural context that promotes a rapid return to pre-pregnancy body shape and appearance, reflecting the emphasis of so-called “bounce-back” culture on quickly restoring the pre-pregnancy body [4,5]. Self-compassion may therefore promote a more accepting and benevolent stance toward postpartum embodiment, allowing women to value their bodies for their functionality and resilience [11].

Perceived compassion from others was also positively associated with body appreciation, underscoring the interpersonal nature of body image experiences in the postpartum period. Compassion from others reflects the perception of being cared for and emotionally supported by significant people in one’s social environment, which may include, but is not limited to, the romantic partner [20]. During postpartum, receiving compassion in the face of bodily vulnerability may help women reinterpret physical changes as legitimate and worthy of care rather than as failures to meet appearance norms. This result is consistent with relational perspectives on body image, which emphasize that body-related evaluations are embedded in social contexts [5,9,10].

Compassion toward others did not show a direct association with body appreciation. A possible explanation is that an outward orientation toward alleviating others’ suffering does not necessarily correspond to a more appreciative relationship with one’s own body. Although compassion toward others is associated with prosociality and emotional regulation, it may be less directly involved in self-directed body evaluations during a period in which the body becomes a central focus of identity and self-perception. Alternatively, the absence of a significant association may also reflect the broad conceptualization of compassion toward others captured by the measure used in the present study, which is not specific to close romantic relationships or postpartum experiences. Moreover, because the expression and interpretation of compassion may be influenced by sociocultural norms, future research conducted across different cultural contexts may help clarify whether these associations vary according to interpersonal expectations and broader cultural values.

Body appreciation was positively associated with couple satisfaction. This finding supports the idea that a more accepting and respectful relationship with one’s body may promote comfort with intimacy and emotional closeness during the postpartum transition. Women who appreciate their bodies may experience less appearance-related inhibition and greater relational security, thereby contributing to higher satisfaction within the couple relationship. This interpretation aligns with evidence that women’s body-related experiences in the postpartum period are closely tied to sexual and relational experiences [9,10] and with emerging findings showing that body appreciation is linked to higher couple satisfaction [17]. Beyond the postpartum context, recent evidence indicates that body appreciation is associated with romantic relationship satisfaction through sexuality-related processes [18], further supporting the relevance of positive body image for couple functioning.

In addition to this indirect pathway, compassion from others showed a direct association with couple satisfaction. This finding highlights the importance of feeling cared for and emotionally supported by significant others during the postpartum period, a time characterized by heightened demands and relational renegotiation [1]. Because compassion from others is not restricted to the romantic partner [20], this association may reflect broader experiences of being emotionally supported, which nonetheless contribute to relational satisfaction within the couple. This direct effect suggests that compassion from others may be related to couple satisfaction through relational mechanisms that do not primarily depend on body-related evaluations.

Importantly, bootstrapping analyses indicated that self-compassion and compassion from others were indirectly associated with couple satisfaction through body appreciation. For self-compassion, this pathway suggests that a kind and non-judgmental attitude toward the self may attenuate appearance-related self-criticism and shame, thereby fostering a more appreciative and respectful relationship with the body. When women perceive their bodies as acceptable and worthy of care, postpartum bodily changes may be experienced as less threatening to the self and to the relationship, supporting greater emotional security within the couple and, in turn, higher couple satisfaction. In this sense, body appreciation may represent an important correlate that statistically accounts for the observed association between self-compassion and couple satisfaction. However, given the cross-sectional nature of the study, this interpretation should not be considered evidence of a causal psychological mechanism.

Regarding receiving compassion from others, the significant indirect association through body appreciation highlights the role of feeling recognized and supported in one’s experiences during the postpartum period in shaping women’s embodied self-experience. When women perceive that others acknowledge their experiences and are motivated to respond with care, this interpersonal experience may be associated with reduced self-criticism and feelings of isolation, creating emotional conditions that are linked to a more accepting and appreciative relationship with one’s own body. In turn, higher body appreciation may be associated with a more secure and less self-threatening engagement within the couple relationship, corresponding to higher couple satisfaction. In this sense, compassion from others showed an indirect statistical association with couple satisfaction through body appreciation, although the cross-sectional design does not allow conclusions regarding the temporal ordering of these associations.

By contrast, the indirect effect of compassion toward others on couple satisfaction through body appreciation was not significant. Although compassion toward others was bivariately correlated with both body appreciation and couple satisfaction, it did not show significant direct or indirect associations in the path model.

Breastfeeding status did not emerge as a significant predictor of couple satisfaction in the model. Although breastfeeding represents a salient embodied and relational experience in the postpartum period [30,31], the present findings suggest that flows of compassion and body appreciation account for a larger portion of the variance in couple satisfaction than breastfeeding status per se. This result should be interpreted cautiously and may reflect the complexity of breastfeeding experiences, which can vary widely in emotional meaning and relational impact.

Taken together, these findings extend the existing literature by showing that compassion flows and body appreciation can be meaningfully integrated to understand couple satisfaction in postpartum women. Importantly, despite the growing interest in compassion flows and positive body image, prior studies have not examined these variables within the postpartum period. The present study therefore contributes novel evidence regarding psychosocial mechanisms that may support couple satisfaction during a critical and understudied life transition.

### 4.1. Limitations and Future Directions

Several limitations of the present study should be acknowledged. First, the cross-sectional and correlational design represents a major limitation of the present study, as it does not allow causal inferences or conclusions regarding the temporal ordering of the observed associations. Although the hypothesized model was theoretically grounded and consistent with the previous literature, mediation analyses based on cross-sectional data should be interpreted as statistical models of associations rather than evidence of an underlying causal process. Rather, they represent statistical models describing patterns of associations that are consistent with the proposed theoretical framework. Accordingly, alternative explanations, including reciprocal or reverse relationships among compassion flows, body appreciation, and couple satisfaction, remain plausible. Future longitudinal and intervention studies are therefore needed to establish temporal precedence and determine whether changes in compassion-related processes are associated with subsequent changes in body appreciation and couple satisfaction.

Second, all variables were assessed through self-report measures, which may be subject to response biases, such as social desirability or shared method variance. This issue may be particularly relevant for constructs related to body image and couple satisfaction, which are sensitive and potentially influenced by normative expectations surrounding motherhood and romantic relationships. Future studies could complement self-report data with partner reports, behavioral indicators, or qualitative approaches to obtain a more comprehensive understanding of how compassion and body appreciation are experienced and expressed in everyday relational contexts.

Third, the study relied on a convenience sample of postpartum women, which may have introduced selection bias and limited the generalizability of the findings. Participants were predominantly Italian, highly educated, married or cohabiting, and primiparous. Consequently, the sample may overrepresent women with greater social, relational, and contextual resources, while underrepresenting postpartum women experiencing more vulnerable circumstances, including those from culturally diverse backgrounds, with lower educational attainment, different family structures, or more complex reproductive histories. Accordingly, the observed associations should be interpreted with caution and may not fully generalize to the broader postpartum population. Additionally, the sample was not selected based on clinical characteristics related to the postpartum period, such as the presence of mood disorders or medical complications. Including more socio-demographically and clinically diverse samples would help determine whether compassion and body appreciation play similar roles across different postpartum populations and levels of psychological or physical vulnerability.

Moreover, breastfeeding was included as a covariate in the model but was operationalized in a relatively broad manner. Breastfeeding experiences are heterogeneous and may differ in emotional meaning, perceived bodily impact, and relational implications. Although breastfeeding status was included to account for a potentially relevant postpartum characteristic, the present study did not collect information regarding important breastfeeding characteristics, such as duration, exclusivity, or women’s subjective experiences. Consequently, it was not possible to examine whether different breastfeeding experiences were differentially associated with body appreciation or couple satisfaction. Future studies could explore whether qualitative aspects of breastfeeding (e.g., perceived burden, bodily comfort, or partner support during feeding) moderate or interact with compassion-based processes and body appreciation in shaping couple satisfaction.

Finally, although the present study focused specifically on body appreciation, other dimensions of positive body image may also be relevant during the postpartum period. For example, future research could examine how body appreciation interacts with body dissatisfaction, sexual self-concept, or perceived bodily functionality in predicting couple satisfaction. Moreover, future studies may benefit from testing more complex analytical models, including potential interaction effects among the different compassion flows and moderated mediation models, to determine whether the associations observed in the present study vary according to the combined influence of different compassion dimensions or other interpersonal and contextual factors. Integrating multiple facets of body image and more complex analytical approaches may help clarify how different dimensions of body image contribute to couple well-being during the postpartum period.

### 4.2. Conclusions

The present study examined the associations between the three flows of compassion—self-compassion, compassion toward others, and compassion from others—body appreciation, and couple satisfaction in postpartum women. By integrating compassion flows with positive body image and couple satisfaction, the findings contribute to a more comprehensive understanding of the psychosocial mechanisms that may support relational well-being during the postpartum transition.

Overall, the results indicate that compassion flows are linked to women’s relationship with their bodies and to couple satisfaction in this sensitive life phase. Self-compassion and compassion from others were associated with higher levels of body appreciation, highlighting the relevance of both intrapersonal and interpersonal forms of compassion for fostering a respectful and accepting view of the postpartum body. Body appreciation, in turn, emerged as a significant correlate of couple satisfaction, suggesting that positive body image may play a central role in facilitating emotional closeness and relational security after childbirth.

Importantly, body appreciation functioned as a psychological mechanism linking compassion flows to couple satisfaction. Self-compassion and compassion from others showed indirect associations with couple satisfaction through body appreciation, suggesting that relating to oneself with care, as well as perceiving that others recognize one’s difficulties and respond with care, may contribute to relational well-being partly through how women experience and evaluate their own bodies. At the same time, compassion from others showed a direct association with couple satisfaction, underscoring the relevance of feeling that one’s experiences are acknowledged and responded to compassionately by significant people in women’s social environment during the postpartum period.

These findings extend the existing literature in several ways. First, they move beyond a sole focus on self-compassion by showing that different compassion flows play distinct roles in postpartum embodiment and relational adjustment. Second, they highlight positive body image, specifically body appreciation, as a key process that was statistically associated with the relationship between compassion flows and couple satisfaction. Third, they provide novel evidence within the postpartum context, a life phase in which bodily changes and couple dynamics are deeply intertwined yet rarely examined together from a compassion-based perspective.

From a practical standpoint, the present findings support the development of postpartum interventions that integrate compassion-based approaches with the promotion of body appreciation. Such programs may incorporate compassion-focused practices aimed at cultivating a kinder and more accepting relationship with oneself, together with strategies designed to foster body appreciation and acceptance of postpartum-related bodily changes. Given the importance of supportive interpersonal experiences during the postpartum transition, selected intervention components may also involve partners or other significant people to strengthen compassionate communication and emotionally supportive interactions. These components could be incorporated into existing postpartum psychological support services and delivered individually, in groups, or through digitally supported formats to increase accessibility for new mothers.

In conclusion, this study underscores the importance of compassion and positive body image as interconnected resources for couple well-being in the postpartum period. By identifying body appreciation as a relevant variable statistically accounting for the associations between compassion flows and couple satisfaction, the present findings provide a theoretically grounded framework for future longitudinal and intervention studies aimed at clarifying the temporal and potentially causal relationships among these constructs. More broadly, these findings support the integration of compassion- and positive body image-based components into postpartum prevention and intervention programs, with the potential to promote both maternal psychological well-being and healthier couple relationships during the transition to parenthood.

## Figures and Tables

**Table 1:** Sociodemographic and perinatal characteristics (*N* = 230).

Variables	Mean ± SD or %
Age	33.65 ± 4.43
Education level	
Primary school	5.5%
Middle school	1.5%
High school	33.3%
Bachelor degree	22.4%
Master’s degree	34.3%
Other	3%
Occupational status	
Full-time employed	63.2%
Part-time employed	17.9%
Homemakers	7%
Unemployed	8.5%
Occasionally employed	3.5%
Marital status	
Unmarried	6%
In a relationship	20.4%
Married/cohabiting	72.6%
Separated/divorced	1%
Nationality	
Italian	98%
Foreign	2%
Place of residence	
Italy	100%
Parity	
Primiparous	92.6%
Multiparous	7.4%
Pregnancy	
Non-premature	90%
Premature	10%
Breastfeeding	
Yes	79.6%
No	20.4%

**Table 2:** Means, standard deviations, and Pearson correlations among study variables (*N* = 230).

Variable	M	SD	1	2	3	4	5
1. Self-compassion	7.22	1.65	—				
2. Compassion toward others	7.76	1.61	0.71 ***	—			
3. Compassion from others	5.96	2.00	0.48 ***	0.35 ***	—		
4. Body appreciation	3.33	0.95	0.46 ***	0.33 ***	0.39 ***	—	
5. Couple satisfaction	2.79	0.93	0.41 ***	0.36 ***	0.49 ***	0.40 ***	—

Note. Values represent means (*M*), standard deviations (*SD*), and Pearson’s correlation coefficients. *** *p* < 0.001.

**Table 3:** Indirect effects of path analysis (*n* = 230).

Predictor → Outcome	Indirect Effect	95% CI (Lower)	95% CI (Upper)	*p* Value
Self-compassion → Couple satisfaction	0.071	0.027	0.159	0.002
Compassion from others → Couple satisfaction	0.043	0.015	0.100	0.003
Compassion toward others → Couple satisfaction	0.000	−0.039	0.046	0.924

## Data Availability

The fully anonymized datasets analyzed during this study are available from the corresponding author upon reasonable request, with no additional privacy or ethical restrictions.
